# How and when screens are used: comparing different screen activities and sleep in Norwegian university students

**DOI:** 10.3389/fpsyt.2025.1548273

**Published:** 2025-03-31

**Authors:** Gunnhild Johnsen Hjetland, Jens Christoffer Skogen, Mari Hysing, Michael Gradisar, Børge Sivertsen

**Affiliations:** ^1^ Department of Health Promotion, Norwegian Institute of Public Health, Bergen, Norway; ^2^ Centre for Evaluation of Public Health Measures, Norwegian Institute of Public Health, Oslo, Norway; ^3^ Alcohol and Drug Research Western Norway, Stavanger University Hospital, Stavanger, Norway; ^4^ Department of Psychosocial Science, University of Bergen, Bergen, Norway; ^5^ WINK Sleep Pty Ltd, Adelaide, SA, Australia; ^6^ Sleep Cycle AB, Gothenburg, Sweden; ^7^ Department of Research and Innovation, Fonna Health Trust, Haugesund, Norway

**Keywords:** screen time, sleep hygiene, social media, sleep, insomnia, young adults

## Abstract

**Introduction:**

Screen use in bed has become a widespread habit, particularly among young people. This behavior has been associated with poor sleep, with some studies indicating that social media use may be especially detrimental. However, there is a scarcity of research directly comparing the relationship between various screen activities and sleep, and most existing studies focus on adolescents rather than young adults. This study aims to explore the relationship between screen use in bed and sleep among students, specifically comparing social media use to other screen-based activities.

**Methods:**

This study utilized data from the cross-sectional Students’ Health and Wellbeing Study of 2022 and included n=45,202 participants aged 18-28 years. Regression analyses were used to assess the relationship between screen time in bed and sleep, comparing social media use with other activities.

**Results:**

A one-hour increase of screen time after going to bed was associated with 59% higher odds of having symptoms of insomnia and a reduction in sleep duration of 24 minutes. The associations between screen time and sleep outcomes did not differ for social media use versus other activities. Independent of screen time, participants who exclusively used social media had lower odds of insomnia and longer sleep duration compared to those engaging in other activities or a mix of activities.

**Discussion:**

The present study found that increased screen time in bed is linked to poorer sleep, across activity type. Future research should refine classifications, assess specific content, and employ experimental approaches to determine causal mechanisms.

## 1 Introduction

Sleep is essential to physical and mental functioning and getting too little sleep or having poor sleep quality negatively impacts mental health, physical health, and even longevity (1–7). By impacting attention span, memory, and other aspects of cognitive functioning, poor sleep can also affect academic performance (8–10), with potentially wide-reaching consequences for individuals’ lives. As a group, students in higher education have been shown to have insufficient sleep duration, falling short of the recommended 7-9 hours (11). Among American students, 36% reported sleeping less than 7 hours per night, while data from Norwegian students show that 30% sleep for 6-7 hours or less (12). Sleep problems beyond short sleep duration are also common, with 22% of males and 34% of females having sleep problems corresponding to the DSM-5 criteria for insomnia, with increasing trends from 2010 to 2018 among Norwegian students (12). Digital media use, including watching TV, gaming, or using social media, has been associated with poor sleep (13–18), and some have suggested that increases in screen time over the past decades may have contributed to a larger proportion of people reporting short sleep times (19). Digital media use has become a part of everyday life, particularly among young people (20, 21). A recent study mapping international trends in screen use found that young adults spend around 12 hours per day using screens (21).

To date, most studies on digital media use and sleep have focused on children and adolescents (16, 22). For these groups, spending a long time on digital media is longitudinally associated with delayed bedtime, increased sleep onset latency, and thus shorter sleep duration – which also overlap with insomnia symptoms, difficulties falling asleep, and daytime sleepiness (15). In line with these findings, interactive and highly stimulating content is discouraged prior to bedtime for children and adolescents (22). Reviews including young adults in addition to adolescents have found similar associations between screen use and sleep (16, 23), but more studies including young adults are needed (22).

Screen use before bed time or in bed is more strongly linked to poor sleep compared to overall screen time during adolescence (16), a pattern also observed among students (14). With the advent of the smartphone, using screens after going to bed has become commonplace, particularly among adolescents and young adults (24, 25). Studies of student populations have shown that over 95% use screens in bed (26, 27). A 2018 study of over 40,000 Norwegian students (the SHOT study) showed a mean total screen time in bed (including a range of screen-based activities) of 46 minutes (14). Furthermore, the continuous tracking of the smartphone use among 815 young adults found that 12% engaged in smartphone activity during their self-reported sleep period (28).

Screen use in bed has been suggested to impact sleep via four routes (29, 30): 1) screen use directly replaces sleep; 2) light exposure suppresses melatonin secretion and delays the circadian rhythm; 3) screen-based activities leads to increased arousal, prolonging the time it takes to fall asleep; and 4) notifications from devices can interrupt sleep after sleep onset. It has been hypothesized that social media may be particularly relevant in terms of increasing arousal, and several researchers have suggested that social media, compared to more “passive” screen use such as watching TV, may be particularly detrimental to sleep (16, 23, 31–33). Furthermore, it may be harder to terminate one’s social media use to go to sleep, due to social expectations, thus delaying bedtime and displacing sleep (34–36). Additionally, notifications from social media may interrupt sleep if the smartphone is left unmuted during the night (37).

Thus, the link between screen use and sleep is likely related to *how* and *when* screens are used, and studies addressing these aspects are increasingly called for (22). However, few studies have directly compared different screen activities, and the evidence for a particularly strong association between social media use and sleep is mixed.

Among adolescents, higher overall screen time was associated with longer sleep onset latency, shorter sleep duration, and more mid-sleep awakenings, and that the associations were stronger for social media use and internet use compared to gaming and TV (32). However, that particular study exclusively asked about gaming on a console or computer and the authors hypothesized that because the smartphone is more readily taken into bed, it may further disrupt sleep, and hence the stronger association for internet and social media use.

Contrary to the hypothesis that social media is more disruptive to sleep than other screen activities, a recent meta-analysis showed that although social media use at one time point predicted worse sleep at a later time point among adolescents, this link was weaker than the link between traditional media use and sleep (15). The authors suggested that social media use, compared to other screen activities, is less disruptive because social media entails socialization, which may be protective in terms of developing sleep problems (15).

Thus, it is unclear whether social media use is worse than other screen activities in terms of disrupting sleep. Most studies to date have focused on total screen time and have not delineated the unique associations of social media use vs other activities at bedtime with sleep. Furthermore, most previous studies on screen time and sleep have involved adolescents and less is known about sleep and screen use among young adults. The aim of this study was to investigate how the amount of time students spend using screens in bed relates to their sleep duration and experience of insomnia, specifically examining the impact of social media use compared to other screen activities, using a large, nationally representative sample.

## 2 Materials and methods

The present study was based on data from the SHOT2022 survey (an acronym for the Norwegian name: Studentenes Helse- og Trivselsundersøkelse [Students’ Health and Wellbeing Study]). The SHOT-survey is a national cross-sectional study of all students enrolled in higher education in Norway. The study contains demographic information as well as several domains of health and lifestyle, including screen use and sleep. The data collection took place from February to April 2022. For the present study, full-time students between 18 and 28 years old were included.

### 2.1 Ethics approval and consent to participate

The study was approved by the Regional Committee for Medical and Health Research Ethics in Western Norway (no. 2022/326437 [SHOT2022]). Written informed consent was obtained electronically after the participants had received a detailed introduction to the study.

### 2.2 Measures

Participants’ age and sex information was derived from their 11-digit Norwegian national identity numbers.

#### 2.2.1 Screen-based activities in bed

Participants were asked to indicate whether they used any electronic media after they had gone to bed for the night. Next, they were asked to indicate which activities they engage in from a list of six activities: 1) Watching movies/TV series, 2) checking social media, 3) surfing, 4) listening to music/audio book/podcast, 5) gaming, 6) reading study related content. As participants could tick several screen activities, a range of combinations of screen activities in bed were possible. A visualization of participants’ responses is available in the supplementary (**Supplementary Figure 1**). For the purpose of the present study, three groups were created based on the activity question: 1) those that only ticked the option “Checking social media” comprised one group called ‘Social media’; 2) those that ticked “checking social media” in addition to other activities were combined into one group, called ‘Social media + other’; and 3) those that did not tick “checking social media” were combined into a group called ‘Non-social media’.

#### 2.2.2 Mean time spent on screen-based activities in bed

The participants indicated how many nights per week (1–7) they used electronic media in bed and approximated how much time they spent each night using a drop-down menu ranging from 5 minutes to “more than 6 hours”. Those indicating that they spent more than 6 hours were included in the 6 hour-group. Mean screentime in bed was calculated by multiplying time spent on the number of nights, divided by seven. Outliers above 3SD from the mean were truncated, meaning that all those scoring above 151.4 minutes (n=596) received a score of 151.4.

#### 2.2.3 Symptoms of insomnia and sleep duration

The participants indicated the number of nights per week they experienced difficulties initiating sleep or maintaining sleep, and the number of days experiencing early morning awakenings or daytime tiredness or sleepiness. Those reporting sleep difficulties were asked to indicate how long the difficulties had persisted. For the purpose of this study and in line with the DSM-5 criteria for insomnia, participants were classified into the insomnia group if they indicated that 1) they had experienced difficulties initiating sleep, maintaining sleep, or early morning awakenings at least three nights per week, 2) they had experienced daytime sleepiness or tiredness for at least 3 days per week, and 3) that these sleep problems had lasted for at least 3 months. The participants also provided detailed self-reported data on their usual bedtime and wake time, recorded separately for weekdays and weekends, with responses indicated in precise hours and minutes. Time in Bed (TIB) was calculated as the difference between the reported bedtime and rise time for each period (weekdays and weekends). To assess Sleep Onset Latency (SOL), participants were asked: “How long does it usually take you to fall asleep (after turning off the lights)?” Responses were recorded separately for weekdays and weekends, measured in hours and minutes. Wake After Sleep Onset (WASO) was assessed with the question: “How long are you awake during the night (after you first have fallen asleep)?” Again, participants provided responses separately for weekdays and weekends, indicating the duration in hours and minutes. See (12) for more details about the included sleep measures.

#### 2.2.4 Missing data

In the target population, there were some missing data on the predictors and outcomes (see **Supplementary Figure 2**). For the sleep variables, 0.3% of the observations were removed as they had invalid responses, including (a) SOL or WASO >12 h, (b) SOL + WASO > TIB, (c) negative values of sleep duration and sleep efficiency. Those with missing data on whether they used screens in bed (0.6%) were excluded, and so were those indicating that they used screens in bed but failed to check any of the screen-based activities (0.3%). Thus, the final population was n=45,654, while the main regression analyses were performed only on those using screens in bed (n=38,809).

#### 2.2.5 Statistical analyses

All analyses were performed using R version 4.1.3 (38) and RStudio version 2023.06.1 + 524 (39). Multiple logistic regression was used to compare those using screens in bed with those not using screens in bed on insomnia and sleep duration, adjusting for age and sex. Multiple logistic regression was used to assess the association between ‘mean screen time in bed’ and ‘insomnia’, and multiple linear regression to assess the association between ‘mean screen time in bed’ and ‘sleep duration’. Both regressions were adjusted for age and sex. In all analyses, a *p*-value of <0.05 indicated statistically significant associations. To assess the influence of type of activity, the base model was compared to a model including type of activity as a predictor and a model also including the interaction term mean time spent×type of activity. The *‘sjPlot’* package in R (40) was used to visualize the relationship between screen time and insomnia and sleep duration for three activity groups.

## 3 Results

All full-time Norwegian students enrolled in higher education in Norway or abroad were invited to participate electronically (n=169,572), with 59,544 completing the web-based questionnaires, resulting in a response rate of 35.1%. For the present study, full-time students between 18 and 28 years old were included (n=46,202). **Table 1** presents descriptive demographic and sleep data separately for men and women. Compared to men, women were slightly younger, a higher proportion used screens in bed, a higher proportion reported symptoms of insomnia and they had a slightly longer sleep duration.

**Table 1:** Descriptives for males and females separately, and for the whole group.

	Women (N=30557)	Men (N=15097)	Total (N=45654)	p value
**Age, mean (SD)**	22.60 (2.22)	22.92 (2.24)	22.70 (2.23)	< 0.001
**Using screens in bed, n (%)**	27159 (88.9%)	12650 (83.8%)	39809 (87.2%)	< 0.001
**Insomnia, n (%)**	11578 (37.9%)	3862 (25.6%)	15440 (33.8%)	< 0.001
**Sleep duration (h), mean (SD)**	7.58 (1.38)	7.53 (1.33)	7.56 (1.36)	0.001

SD, Standard deviation.

Among those not using screens in bed, the proportion reporting symptoms of insomnia was 31.1% and sleep duration was 7.56 (SD 1.37). In comparison, the proportion reporting symptoms of insomnia was 34.4% and sleep duration was 7.57 (SD 1.33) among those using screens. Regression analyses with screen use (yes/no) as the predictor, adjusted for age and sex, showed that those not using screens in bed had 24% lower odds of reporting symptoms of insomnia (OR = 0.86, 95CI 0.81-0.92, p<.001). The difference between screen users and non-screen users was not significant for sleep duration (B = 0.02, SE = 0.02, p=.299).

Further regression analyses were performed after excluding those not using screen-based activities in bed, as the predictor was time spent on screen-based activities. **Table 2** provides a description of the groups. Among the young adults who used screens in bed, the majority (69%) used social media in addition to other screen-based activities (mixed group); the most common other screen activities being internet surfing and watching movies. The social media and the non-social media groups both accounted for around 15%. In the non-social media group, watching movies or TV-series or listening to audio books were the most common activities. **Supplementary Figure 1** shows the number of participants ticking only one activity and the 10 most common combinations of activities. Of the 10 most common combinations, nine included using social media. While those only using social media constituted the largest group (n=6103), all six consecutive groups in terms of size (n ranged from 4051 to 1966) were different combinations of social media with one or two other activities.

**Table 2:** Descriptives for the screen activity groups.

		Social media (N=6103)	Social media + other (N=27558)	Non-social media (N=6148)	Total (N=39809)	p value
**Screen activities**	**Movies/TV series, n (%)**	0 (0.0%)	15422 (56.0%)	2963 (48.2%)	18385 (46.2%)	< 0.001^1^
	**Checking social media, n (%)**	6103 (100.0%)	27558 (100.0%)	0 (0.0%)	33661 (84.6%)	< 0.001^1^
	**Surfing, n (%)**	0 (0.0%)	15626 (56.7%)	2312 (37.6%)	17938 (45.1%)	< 0.001^1^
	**Music/audio book, n (%)**	0 (0.0%)	14171 (51.4%)	2867 (46.6%)	17038 (42.8%)	< 0.001^1^
	**Gaming, n (%)**	0 (0.0%)	4842 (17.6%)	629 (10.2%)	5471 (13.7%)	< 0.001^1^
	**Study, n (%)**	0 (0.0%)	5630 (20.4%)	775 (12.6%)	6405 (16.1%)	< 0.001^1^
	**Sex**					< 0.001^1^
	Women, n (%)	5065 (83.0%)	18925 (68.7%)	3169 (51.5%)	27159 (68.2%)	
	Men, n (%)	1038 (17.0%)	8633 (31.3%)	2979 (48.5%)	12650 (31.8%)	
	**Age**					< 0.001^2^
	Mean (SD)	22.54 (2.10)	22.54 (2.17)	23.20 (2.38)	22.64 (2.21)	
	**Nmb. Evenings using screens**					< 0.001^2^
	Mean (SD)	6.29 (1.24)	6.43 (1.09)	5.96 (1.43)	6.34 (1.19)	
	**Nmb. of screen activities**					< 0.001^2^
	Mean (SD)	1.00 (0.00)	3.02 (1.08)	1.55 (0.76)	2.48 (1.25)	
	**Time spent on screens in bed, minutes**					< 0.001^2^
	Mean (SD)	27.88 (24.80)	45.74 (37.56)	38.77 (37.60)	41.95 (36.49)	
	**Insomnia, n (%)**	1684 (27.6%)	9715 (35.3%)	2280 (37.1%)	13679 (34.4%)	<0.001^1^
	**Sleep duration, hours**					
	Mean (SD)	7.76 (1.25)	7.56 (1.36)	7.38 (1.47)	7.56 (1.37)	<0.001^2^

^1^Pearson’s Chi-squared test.

^2^Linear Model ANOVA.

**Figure 1:**
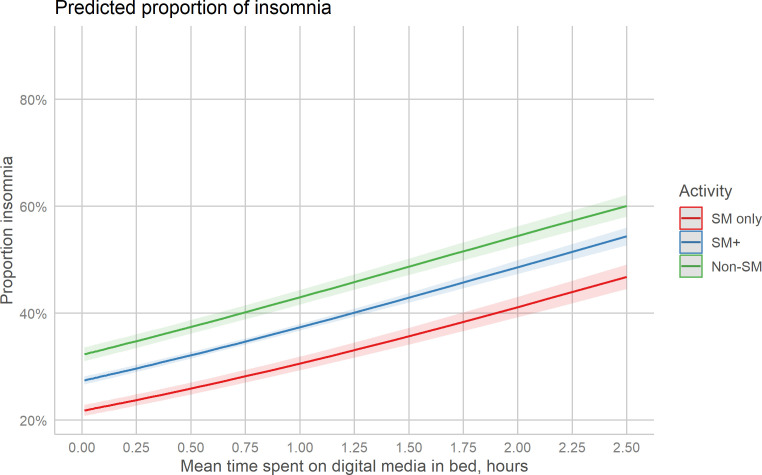
Predicted probabilities of insomnia across time screen time, separately for three activity groups. SM, Social media.

The interaction between time spent and activity group was not significant, suggesting that the association between time spent and symptoms of insomnia was similar across groups (**Table 3**). AIC did not improve by adding the interaction term and model 1 was chosen as the final model. The model showed that there was a significant positive association between ‘mean screen time in bed’ and ‘insomnia’, where the odds of reporting symptoms of insomnia rose by 59% for each 1-hour increase in screen time (OR = 1.59, *p*<.001), holding all other variables constant. Compared to using only social media, engaging in other activities in addition to social media was associated with a higher odds of reporting symptoms of insomnia of 35% (OR = 1.35, *p*<.001), holding all other variables constant. For the non-social media group, the association with symptoms of insomnia was 1.71 (*p*<.001). **Figure 1** shows the proportion reporting symptoms of insomnia plotted against time spent on screen activities in bed for each activity group.

**Table 3:** Binominal regression model, insomnia as outcome variable.

Variables	Base model			Model 1			Model 2		
	OR	95CI	p	OR	95CI	p	OR	95CI	p
Mean time spent, hours	1.625	1.568-1.683	<.001	1.586	1.530-1.644	<.001	1.664	1.458-1.902	<.001
Sex: Man	0.566	0.540-0.594	<.001	0.534	0.509-0.560	<.001	0.534	0.509-0.561	<.001
Age	1.033	1.023-1.043	<.001	1.028	1.028-1.038	<.001	1.028	1.018-1.038	<.001
Activity: SM+other				1.354	1.270-1.443	<.001	1.372	1.246-1.511	<.001
Activity: Non-SM				1.711	1.579-1.853	<.001	1.849	1.643-2.081	<.001
Mean time spent×SM+other							0.966	0.840-1.109	.623
Mean time spent×Non-SM							0.878	0.748-1.029	.108

AIC, base model: 49014, model 1: 48842, model 2: 48842. SM, Social media.

**Figure 2:**
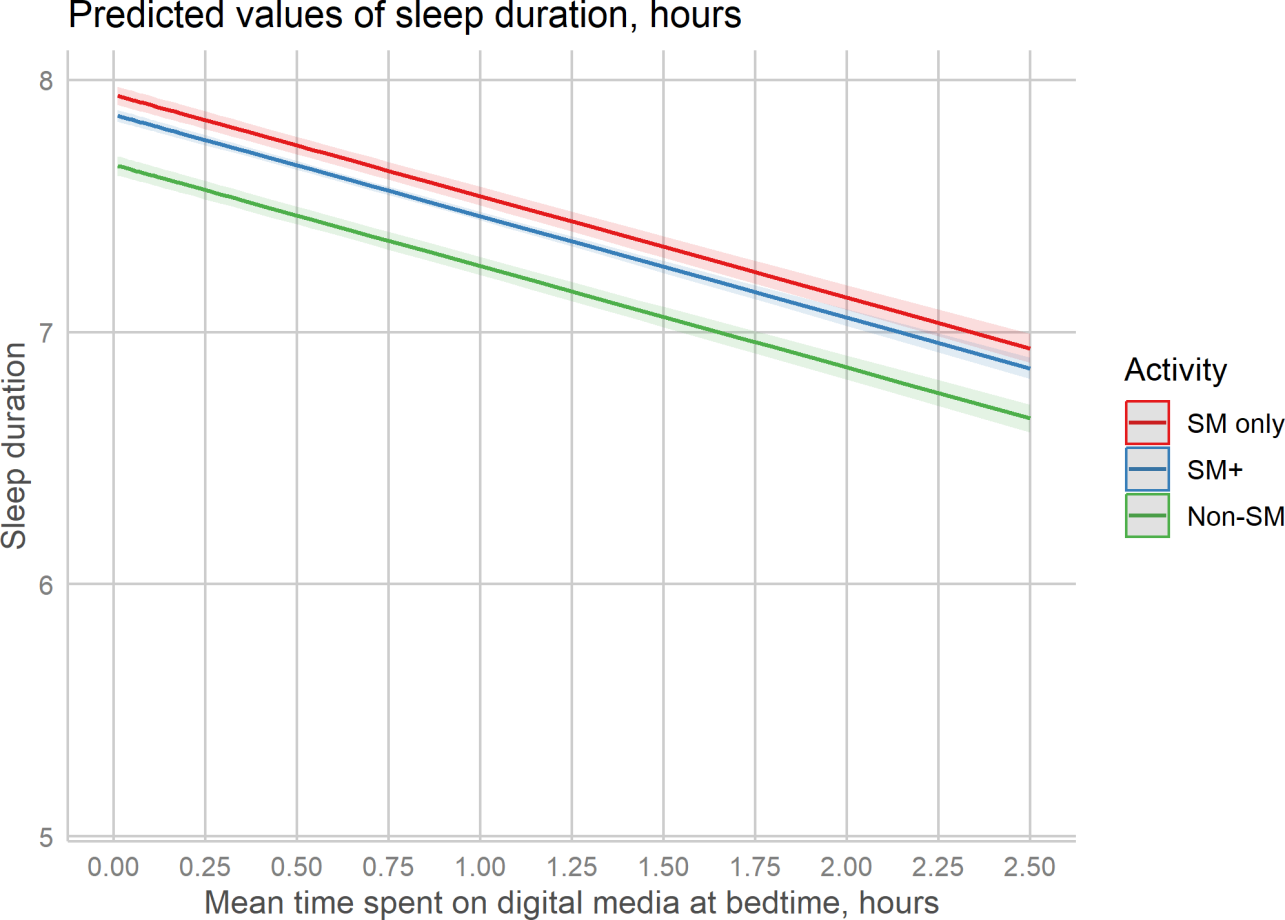
Predicted sleep duration across time screen time, separately for three activity groups. SM, Social media.

For sleep duration, the interaction between time spent and activity group was not significant, suggesting that the association between time spent and sleep duration was similar across activity groups (**Table 4**). The model with the interaction term had a poorer fit, and model 1 was chosen. Model 1 showed a significant association between ‘mean screen time in bed’ and ‘sleep duration’ (B= -0.402, p<.001), suggesting a negative association between screen time and sleep duration where a 1-hour increase in screen time is associated with a decrease of sleep duration of 0.40h = 24 minutes, holding all other variables constant. Compared to using only social media, other activities in addition to social media were associated with decrease of sleep duration of 4.7 minutes, holding all other variables constant. For the non-social media group, the decrease in sleep duration was 16.7 minutes. **Figure 2** shows mean sleep duration plotted against time spent on screen activities in bed for each activity group.

**Table 4:** Multiple linear regression models, sleep duration as outcome.

Variables	Base model			Model 1			Model 2		
	β	SE	p	β	SE	p	β	SE	p
Mean time spent, hours	-0.407	0.012	<.001	-0.402	0.012	<.001	-0.384	0.043	<.001
Sex: Man	-0.060	0.015	<.001	-0.029	0.015	.055	-0.029	0.015	.056
Age	-0.030	0.003	<.001	-0.027	0.003	<.001	-0.027	0.003	<.001
Activity: SM+other				-0.079	0.020	<.001	-0.075	0.030	<.05
Activity: Non-SM				-0.278	0.026	<.001	-0.250	0.037	<.001
Mean time spent×SM+other							-0.012	0.045	.776
Mean time spent×Non-SM							-0.048	0.051	.348

AIC, base model: 128626, model 1: 128496, model 2: 128499. SM, Social media.

## 4 Discussion

In this study, we assessed the association between screen time after going to bed for the night, and sleep, and delved into comparisons between social media use and other screen activities. Overall, the results show that more screen time in bed is associated with a higher likelihood of reporting symptoms of insomnia and shorter sleep duration. Specifically, a 1-hour increase in screen time after going to bed was associated with an increase in the odds of reporting symptoms of insomnia of 59% and a reduction of sleep duration of 24 minutes. In terms of screen activities, it was most common to use social media in combination with other screen activities, with 69% of the participants doing so. Around 15% of participants only used social media and another 15% reported not using social media. Independent of screen time, there were differences in the proportion of participants reporting symptoms of insomnia and differences in the mean sleep duration across activity groups. Surprisingly, the group only using social media had the lowest proportion reporting symptoms of insomnia and the longest sleep duration, while the group only engaging in other activities besides social media had the highest proportion reporting symptoms of insomnia and the shortest sleep duration. Those using social media in addition to engaging in other activities were in between the two other groups. The association with screen time was not significantly different for the activity groups. Those not using screens in bed had 24% lower odds of reporting symptoms of insomnia, while there was no difference between screen users and non-users in terms of sleep duration.

Our finding of a similar association between screen time and sleep across screen activities contrasts with some previous work, where one study found a stronger association between social media use and sleep as well as internet use and sleep, compared to other activities (32), while one meta-analysis suggested a weaker association for social media use (15). The study by Hisler et al. (32) differed from the present study in several ways, which may explain the discrepancy between the results. Firstly, their study included adolescents and not young adults, and it is possible that the association between different screen activities and sleep varies across age groups. For example, the heightened sensitivity to socio-emotional cues in adolescence compared to other life phases (41) might result in more arousal and subsequent sleep problems (and a stronger association between social media use and sleep) among adolescents compared to older age groups. Second, Hisler et al. (32) measured overall screen use, while the present study focused on screen use after going to bed for the night. As highlighted by Hisler and colleagues, their finding of a stronger link between sleep and social media/internet use may have been influenced by the device used (smartphones), which are easier to bring into bed compared to gaming consoles and computers. Thus, their findings may be related to the timing and location of the screen use (in bed at bedtime), rather than the specific activity. The meta-analysis by Pagano et al. (15) showed that the association between social media use and later sleep problems was weaker for social media versus other screen activities. Although we did not find that the association differed by activity, we found that those only using social media in bed on average had better sleep than those engaging in other activities. One interpretation of this finding is that exclusively using social media compared to a combination of social media and other activities or only other activities signals a preference for socializing, which may reflect being part of a social network and an interest in maintaining social relationships, which, in turn, is protective to poor sleep (42).

Another interpretation is that social media use is not the preferred activity for students who struggle the most with their sleep. Some may even use technology as a sleep aid (30), and other activities than social media use may be more used for this purpose, such as watching a TV series or listening to music. Watching TV series/movies and listening to music, podcasts or audio books were endorsed by high percentages in both the mixed and non-social media groups, perhaps reflecting that many students in these groups used technology as a sleep aid or as a past-time while waiting for sleep to “happen” (43). Furthermore, not using social media in bed might be a deliberate strategy to alleviate existing sleep problems, rooted in own experiences of social media disrupting sleep or in beliefs that social media use disrupts sleep, which might partly explain why being in the non-social media group was associated with the worst sleep. This interpretation may also apply to those not using screens in bed; it could be a deliberate strategy to alleviate existing sleep problems, explaining the relatively small difference between the screen users and non-screen users in terms of sleep.

In terms of mechanisms, our results can be interpreted as supporting the displacement hypothesis, i.e., that the time spent on screens displaces sleep. If increased arousal was an important contributor to poorer sleep, we would expect to see different associations between screen time and sleep for the different screen activities. In support of this interpretation, a recent theoretical review pointed to displacement as the most important mechanism for the association between technology use and sleep, highlighting that light exposure and increased arousal have shown minimal effects on sleep in experimental studies (30). A recent study on young adolescents demonstrated this point by showing that all forms of screen use were associated with extended shuteye latency (i.e., a longer time between going to bed and attempting to sleep), but not with sleep onset latency (i.e., the time from attempting to sleep to sleep onset) or waking up after going to sleep (44). The authors interpreted this as demonstrating that the association between screen time in bed and sleep is largely due to displacement, and not arousal and/or light exposure. Importantly, the screen activities measured in the present study were very broad and each activity may have involved very different experiences, and we are unable to rule out a role of arousal based on the present finding.

Furthermore, we are unable to infer causality based on cross-sectional data. The association between time spent on screen activities and sleep may, as mentioned, be based on the fact that those who struggle with sleep use screens as a past-time or a sleep aid. For example, in the study by Hisler and colleagues (32), the association between sleep and gaming was rendered non-significant when controlling for chronotype, suggesting that the association was explained by late chronotype adolescents (‘owls’) filling the time they would already be awake with gaming. For social media and internet use, the associations became weaker when controlling for chronotype, but were still significant, suggesting that the association was only partly explained by chronotype and filling the time spent awake. One experience sampling study following student participants over 7 consecutive days, found no negative effect of screen time in the two hours before falling asleep on sleep duration or sleep quality (45), suggesting that the causal effect is much smaller than the associations found in cross-sectional studies such as the current one. Importantly, that study measured screen use in the two hours before falling asleep and not specifically screen use after going to bed. This means that their participants did not necessarily use screens close to bedtime, which may, at least in part, explain why they found no relationship. Interestingly, Sumter et al. (45) also found that using social media platforms in the two hours before falling asleep was positively related to sleep quality.

Our results should be interpreted with caution given that different screen activities were categorized together. Importantly, gaming was combined with other screen activities such as watching TV series, listening to music/podcasts and reading study related material. Gaming has been linked to worse sleep in some studies (46, 47) and may have contributed to the higher proportion reporting symptoms of insomnia and shorter sleep duration in the comparison groups. Unfortunately, too few participants engaged only in gaming to allow us to separate this group from the others. Furthermore, each of the screen activities included in the present study may vary greatly. Social media is highly heterogenous, both in terms of the platforms used, but also in terms of the specific content and the interactions individuals have on social media. It is possible that negative interactions or experiences, such as bullying or being exposed to upsetting content, will have a negative impact on sleep, while positive social interactions and feeling connected to others would support sleep. Focusing on the specific behaviors and experiences of individuals using screens in bed may provide further insight into the relationship between screen use and sleep.

### 4.1 Implications and further research

The current findings suggest a role of screen use in reduced sleep duration and quality among students, but other research approaches are needed to elucidate the direction of effects. Importantly, there was no evidence for social media use being worse than other screen activities in terms of disrupting sleep. According to these findings, recommendations to avoid certain screen activities over others at bedtime may not be warranted. Instead, public health messages could aim at reducing the overall time spent on screen activities, which broadly seems to be a reasonable recommendation. However, there are probably large individual differences in how screen use relates to sleep, with some young people using screens as a way to cope with sleep problems (43). To elucidate how, why, and for whom screen activities disrupt sleep, follow-up studies tracking participants over time in natural settings are needed. Importantly, the present study included students from a Western culture. A recent meta-analysis found that the association between social media use and sleep was stronger in Eastern compared to Western cultures (48), and the current findings may not be generalized to other countries or cultures.

### 4.2 Strengths and limitations

An important strength of the present study is the large sample size, allowing for a comparison of groups engaging in different screen activities. Furthermore, the study focuses on a student population, whereas most previous studies on screen use and sleep focus on adolescents. Additionally, it is one of the few studies comparing different screen activities in terms of their association with sleep. The study also has some important limitations. First, the data are cross-sectional and only demonstrates a relationship between screen time and sleep, and not causal effects. Second, the moderate response rate of 35.1%, which is somewhat low for web-based surveys among students in this age group (49), is a limitation of the study. Third, the measures of sleep and screen use are based on self-report, which may involve bias, and the sleep measures lacks clinical assessment and objective measurement through polysomnography or sleep diaries. The findings should be interpreted with these limitations in mind. Forth, we used broad categories of screen activities that each could potentially entail a wide range of exposures and experiences. Fifth, we did not consider nighttime screen use after sleep onset. Having multiple social media accounts and being highly active on social media may entail more over-night notifications disrupting sleep. Overnight disruptions from smartphones or other devices are an understudied area that needs research attention (30).

## 5 Conclusion

This study explored the association between screen time in bed and sleep, comparing social media use to other screen activities among almost 40,000 university students. The results showed that increased screen time in bed correlates with a higher likelihood of reporting symptoms of insomnia and shorter sleep duration, whereby each additional hour of screen time was linked to a 59% increase in insomnia risk and 24 minutes less sleep. There was no difference in the association between screen time and sleep for social media versus other screen activities, suggesting that social media may not be considered worse than other screen activities in terms of disrupting sleep. Irrespective of screen time, those only using social media had the lowest proportion reporting symptoms of insomnia and the longest sleep duration, while those who only engaged in other screen activities had the worst sleep.

Future research should address the limitations of the current study by exploring screen activities in more detail, examining specific experiences and interactions related to screen use, and considering potential overnight disruptions due to device notifications. Longitudinal studies tracking screen use and sleep in naturalistic settings would also be beneficial for understanding causal relationships. Together, such efforts could clarify the impact of bedtime screen use on sleep and inform targeted recommendations for students and other populations.

## Data Availability

The data analyzed in this study is subject to the following licenses/restrictions: Norwegian data protection regulations and GDPR impose restrictions on sharing of individual participant data. However, researchers may gain access to survey participant data by contacting the publication committee. Approval from the Norwegian Regional Committee for Medical and Health Research Ethics (https://rekportalen.no/) is a pre-requirement for access to the data. The dataset is administrated by the NIPH, and guidelines for access to data are found at https://www.fhi.no/en/more/access-to-data. Requests to access these datasets should be directed to BS, borge.sivertsen@fhi.no.
