# Supplementary material for: How and when screens are used: comparing different screen activities and sleep in Norwegian university students
Source: Front Psychiatry. 2025 Mar 31;16:1548273. doi: 10.3389/fpsyt.2025.1548273 (PMC12015757; doi:10.3389/fpsyt.2025.1548273)
Supplement: Supplementary file 1 [file SupplementaryFile1.docx]

Supplementary Material

# Supplementary Figures and Tables

## Supplementary Figures


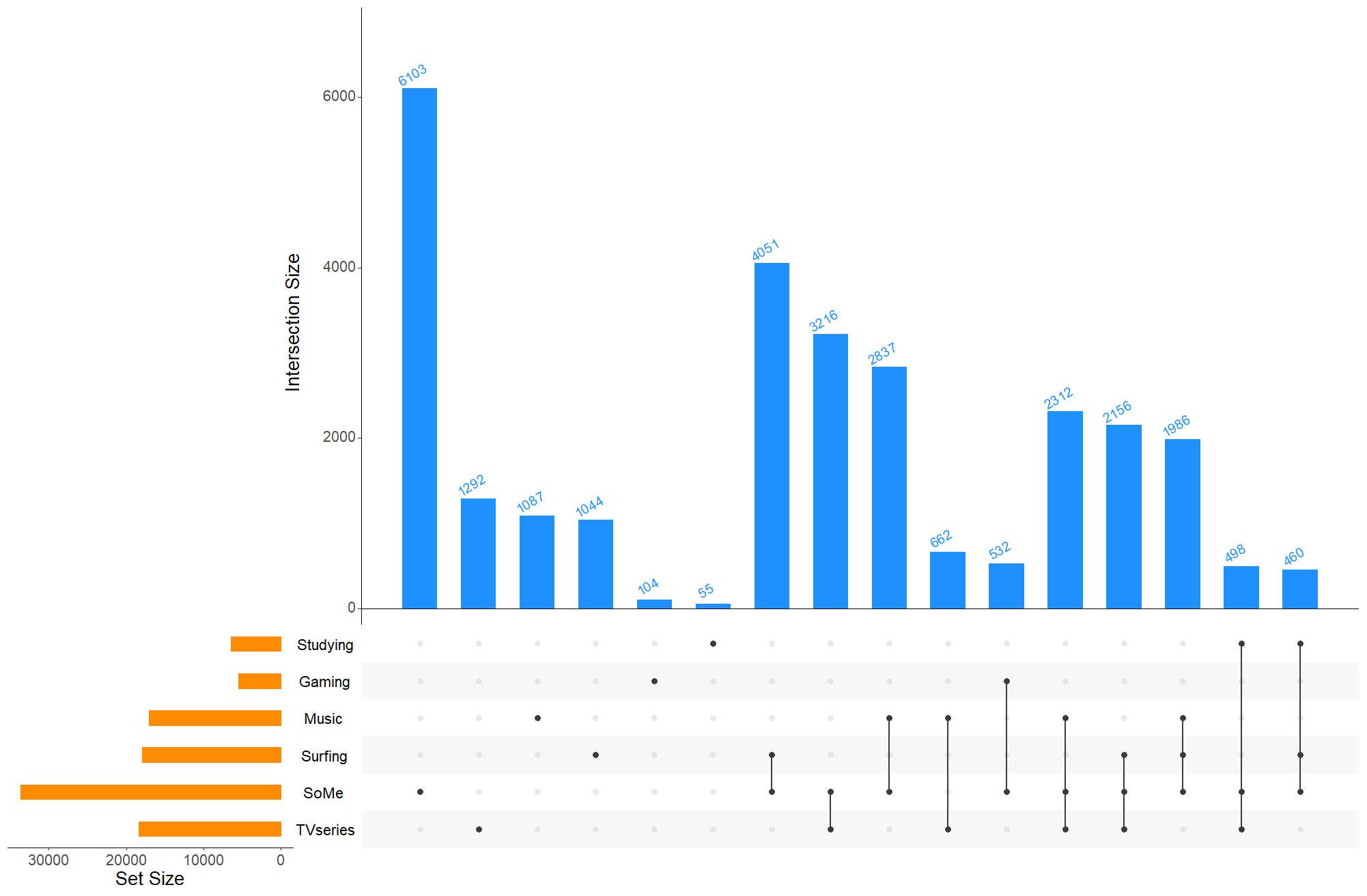


Supplementary Figure 1. Plot showing the total n for each activity (horizontal orange bars), the n for only one activity (intersections; the six first vertical blue bars), and the ten most frequent activity combinations (intersections; the ten following vertical blue bars). The black dots indicate the relevant activities for each intersection. Created using the *‘UpSetR’* package for R.


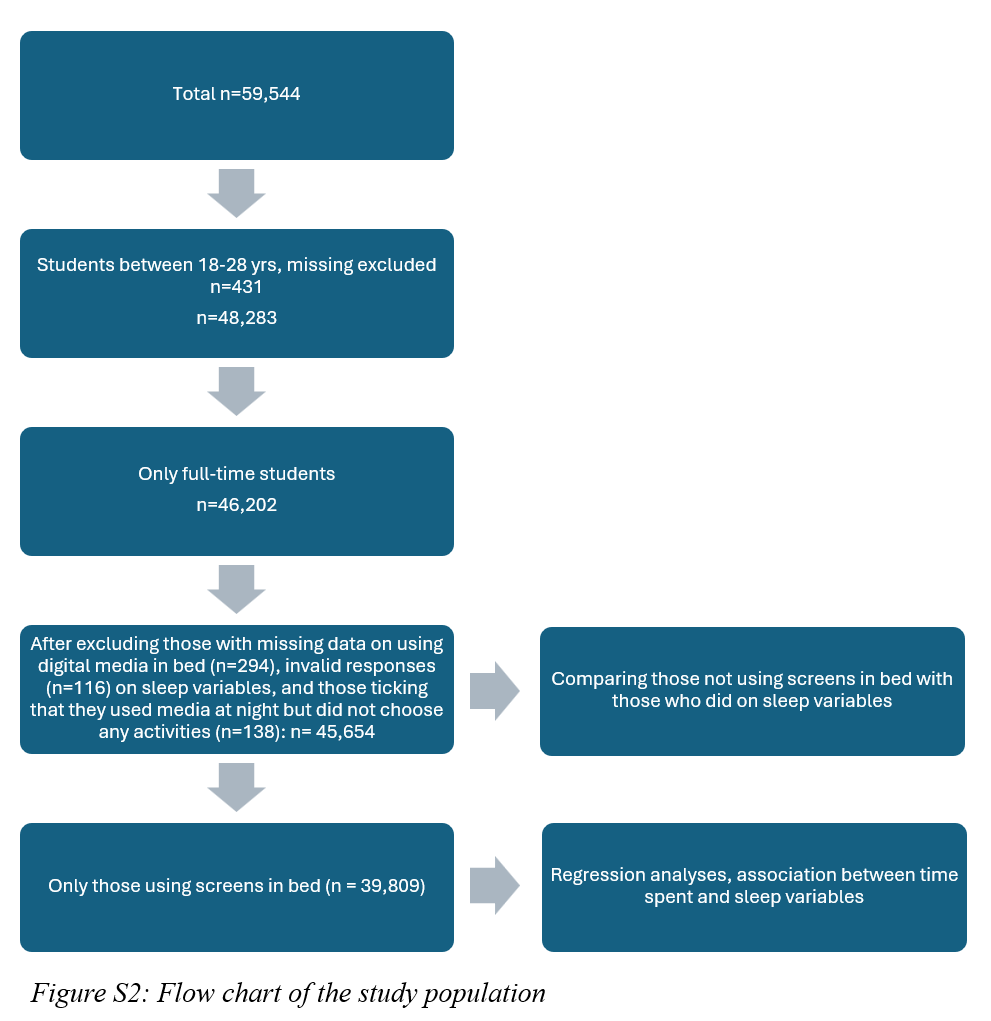


**Supplementary Figure 2.** Flow chart of the study population.
